# Warning Labels as a Public Health Intervention: Effects and Challenges for Tobacco, Cannabis, and Opioid Medications

**DOI:** 10.1146/annurev-publhealth-060922-042254

**Published:** 2024-04-03

**Authors:** Lucy Popova, Zachary B. Massey, Nicholas A. Giordano

**Affiliations:** 1 School of Public Health, Georgia State University, Atlanta, Georgia, USA; 2 School of Journalism, University of Missouri, Columbia, Missouri, USA; 3 Nell Hodgson Woodruff School of Nursing, Emory University, Atlanta, Georgia, USA

**Keywords:** tobacco, cannabis, opioid medications, warning labels

## Abstract

Warning labels help consumers understand product risks, enabling informed decisions. Since the 1966 introduction of cigarette warning labels in the United States, research has determined the most effective message content (health effects information) and format (brand-free packaging with pictures). However, new challenges have emerged. This article reviews the current state of tobacco warning labels in the United States, where legal battles have stalled pictorial cigarette warnings and new products such as electronic cigarettes and synthetic nicotine products pose unknown health risks. This article describes the emerging research on cannabis warnings; as more places legalize recreational cannabis, they are adopting lessons from tobacco warnings. However, its uncertain legal status and widespread underestimation of harms impede strict warning standards. The article also reviews opioid medication warning labels, suggesting that lessons from tobacco could help in the development of effective and culturally appropriate FDA-compliant opioid warning labels that promote safe medication use and increased co-dispensing of naloxone.

## 1. INTRODUCTION

Consumer products should ideally be harm free. However, many of these products involve health risks. Warning labels are messages attached to a product or packaging that communicate the potential dangers of the product when these dangers cannot be eliminated through product design (87). Warnings should, at a minimum, include a “signal word” (such as “Warning!”), a description of the nature of the hazard and/or consequences (what will happen if the warning is not followed), and instructions (what to do to reduce or eliminate the hazard) (90). How these pieces of information are presented to maximize the effectiveness of the warnings has been researched extensively across various disciplines, from human factor design and engineering to communication and public health. This article focuses on warning labels on three types of substances: tobacco (here, commercial and not traditional or ceremonial tobacco), cannabis, and opioid medications.

These three products are similar in that they have inherent health risks, but they differ in the benefits and harms that need to be communicated to potential or existing users. Tobacco, particularly its most prevalent method of use—smoking—is the leading cause of preventable death and disease globally and has no medical benefits. Despite potential health benefits (e.g., reduced nausea for cancer patients) and increasing public opinion that cannabis is low risk, cannabis use can also have negative health impacts. Opioid medications are approved drugs, although they have significant potential for abuse. Thus, the purposes of warning labels on these three products differ along the continuum from deterrence (tobacco) to informed use (opioids), with cannabis arguably somewhere in between. The following sections review the history of warnings for each product and summarize research and the current state of warnings, focusing on the United States but adding global examples as appropriate.

## 2. TOBACCO PRODUCTS

### 2.1. Cigarette Warning Labels

In the second half of the twentieth century, as evidence of the harmful health effects of smoking accumulated, calls for warning labels on cigarettes began to emerge. Although text warning labels in the United States were first proposed as early as 1957 and pictorial warnings (in the form of a skull and crossbones) as early as 1959 (75, 102, 103), the warnings were not enacted into law until 1965. They first appeared on the side of the cigarette packs in 1966.

The tobacco industry played a major role in delaying and weakening the warnings. In the United States, the originally proposed warnings mentioned specific diseases (cancer and heart disease) and death (104). Through political pressure, the industry successfully reduced the initial warning to “Caution: Cigarette Smoking May Be Hazardous to Your Health.” Concurrently, the tobacco industry successfully lobbied for local or state governments to be prohibited from passing any laws related to warnings on cigarette packaging or advertising (12). Similarly, in many other countries, the tobacco industry delayed the implementation of warning label policies. The threat of litigation was sometimes enough to delay the implementation of strengthened warnings by several years, as with the New Zealand plain packaging law, which increased the size of the warnings and required brand colors and logos to be removed from the packs and replaced with a single color (19).

Cigarettes in the United States currently carry one of four warning labels, which were last updated in 1985. The 2009 Family Smoking Prevention and Tobacco Control Act (TCA) gave the US Food and Drug Administration (FDA) authority to regulate tobacco products; it also established the text of nine new warning labels and tasked the FDA to develop pictorial warnings to accompany them. In the TCA, the new labels were required to cover 50% of the front and back of cigarette packs and 20% of advertisements. The FDA complied and developed the first nine pictorial warnings slated to appear on cigarette packs in 2012. However, tobacco companies sued, and, in two different cases, the court upheld the FDA’s authority to create warning labels (25) but struck down these specific labels (86). Legal and public health literature describes the nuances of these cases and criticizes the decisions (21, 61, 101). The FDA decided not to appeal the ruling but to develop a new set of warnings, which it eventually did in 2020 (spurred by a lawsuit from public health and medical organizations and individual doctors). The new set of 11 warnings was supposed to go into effect in 2021, but tobacco companies sued again, and a federal judge first postponed the implementation multiple times and eventually ruled that this set of warnings also violated tobacco companies’ First Amendment rights. It was a partial ruling, similar to two earlier cases in which the specific warnings were struck down; however, this time, the court declined to rule on the constitutionality of the FDA’s overall authority to require pictorial warning labels. On February 1, 2023, the FDA appealed the district court’s decision to the Fifth Circuit (84).

The legal saga regarding pictorial warnings on cigarettes in the United States continues. Mean-while, other countries have introduced increasingly large warning labels that spread from the side of the pack to the front and the back of the pack and are subsequently required to fill an increasingly higher percentage of the pack’s surface: 30%,50%,80%,and even 90%.Iceland was the first to introduce pictorial warnings in 1985 (49). Australia in 2012 and 15 other countries since then (70) began requiring plain or standardized packaging. This is the latest stage in the evolution of warning labels, where large warnings appear on packs from which the brand colors and logos have been removed, and all packs, regardless of brand, come in a single color (frequently, drab olive).

**Figure 1:**
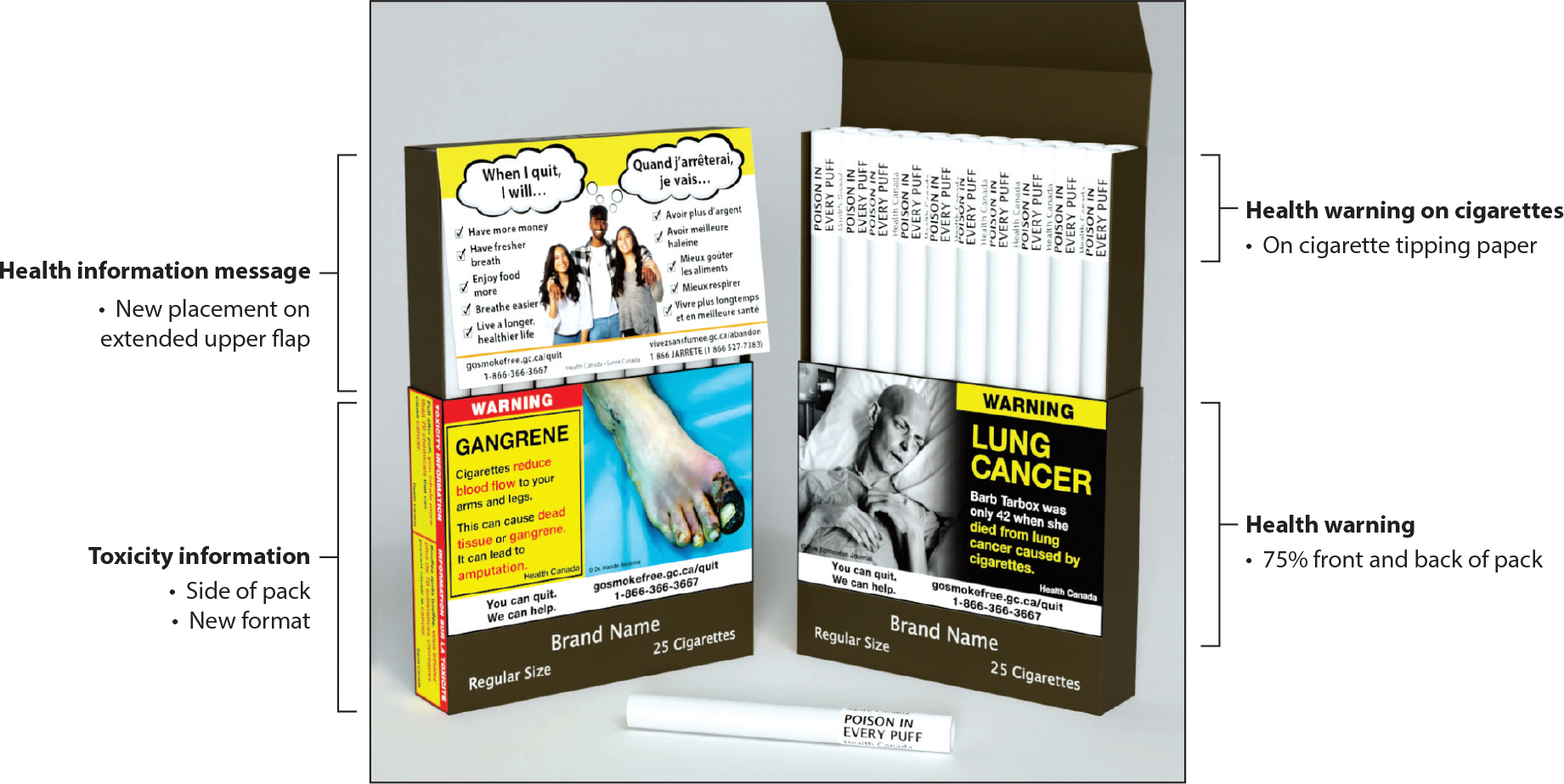
Proposed warning labels on cigarette packs and cigarette sticks, Canada. Figure adapted with permission from Health Canada.

In addition to warnings on the outside of the pack, some countries have used inserts or onserts (small pieces of paper packed inside the pack or under the cellophane on the outside of the pack) to communicate warning messages. Further innovations have been proposed, such as putting warnings on cigarette sticks themselves, intended to reduce the appeal of cigarettes and motivate quitting (47, 50). Canada is implementing a policy to put warnings on individual cigarettes (starting in 2024), the first country to do so (Figure 1).

### 2.2. Warnings on Alternative Tobacco Products

As smoking rates declined in the United States and other high-income countries, tobacco companies introduced novel tobacco products, such as electronic cigarettes (e-cigarettes, also called vapes), snus, dissolvables, and, more recently, heated tobacco products and nicotine pouches. To avoid FDA regulation, some of the products (predominantly e-cigarettes and nicotine pouches) have included claims that they contain synthetic instead of tobacco-derived nicotine, and some have modified the warning labels to advertise that claim (63). In 2022, the US Congress extended the FDA’s authority to cover synthetic nicotine to address this latest marketing tactic (98).Because these alternative tobacco products are new and their long-term health harms are largely unknown, the required US government warnings have been limited to informing users about the presence of nicotine, such as “WARNING: This product contains nicotine. Nicotine is an addictive chemical.” Canada introduced a warning that more directly addresses health harms: “WARNING: Vaping products release chemicals that may harm your health.” In addition, several jurisdictions (Israel, the Netherlands, and the Canadian province of British Columbia) require plain packaging for e-cigarettes (70).

Cigarettes are a lethal consumer product that kills half of its users; noncombustible alternative tobacco products expose users to lower amounts of some harmful chemicals and are likely to reduce harm to smokers if smokers switched to alternative tobacco products completely. Thus, there have been calls to put this information (that specific products are less harmful than cigarettes) on warning labels for alternative tobacco products. For example, studies have examined replacing e-cigarette warning labels with comparative risk messages (18), such as “Use of this product is much less harmful than smoking” (59). This comparative risk message was found to reduce perceptions of harm and addictiveness of e-cigarettes in both smokers and nonsmokers (59). In 2011, R.J. Reynolds filed a citizen petition requesting the FDA to allow a change in the language for one of the smokeless tobacco warnings from “WARNING: This product is not a safe alternative to cigarettes” to “WARNING: No tobacco product is safe, but this product presents substantially lower risks to health than cigarettes.” The FDA denied this petition, stating that the current warning is factual and not misleading and that there was not enough evidence that the proposed warnings would promote a greater public understanding of the harms of smokeless tobacco.

In the United States, companies that want to advertise their products as less harmful than other products (usually cigarettes) can apply for a modified risk (or modified exposure) claim authorization. The FDA has approved several such applications, for example, a modified risk claim for General Snus (“Using General Snus instead of cigarettes puts you at a lower risk of mouth cancer, heart disease, lung cancer, stroke, emphysema, and chronic bronchitis”) and a modified exposure claim for a heated tobacco product IQOS (e.g., “Scientific studies have shown that switching completely from conventional cigarettes to the IQOS system significantly reduces your body’s exposure to harmful or potentially harmful chemicals”). However, it should be noted that these claims are not meant to replace the warnings; all these products still carry the required warnings.

Recent studies in multiple countries have examined relative risk messages. Most of this research focused on e-cigarettes. A systematic review of quantitative studies concluded that relative risk messages reduced the perceived harm of e-cigarettes and sometimes increased smokers’ intentions to use e-cigarettes (27). However, multiple challenges of communicating relative risk were revealed, particularly in qualitative studies. Specifically, smokers interpreted unknown risks of e-cigarettes as potentially worse than the known risks of cigarettes; they did not always clearly understand what “switching completely” meant and were skeptical about the reduced risk claims and message source (81, 107).

In summary, warning labels on cigarettes have been extensively studied both in experimental settings (7, 78) and in the real world (77); these studies show that large pictorial warnings in combination with plain packaging are effective at increasing harm perceptions and changing behavior. The evolution of alternative tobacco products requires continuous research on how to best communicate their harms, both absolute and relative to the most dangerous consumer product, cigarettes. This research has informed warnings on other substances, such as cannabis.

## 3. CANNABIS

Nonmedical recreational cannabis is becoming increasingly available as a retail consumer product in North America. Canada legalized recreational cannabis nationally in 2018 (36). In the United States, as of 2023, recreational cannabis was allowed in 23 states and the District of Columbia (39, 41, 45, 110). Legalization of recreational cannabis represents an important shift in drug policy with implications for consumer health.

With the legalization of cannabis, consumers in the United States increasingly view cannabis as not very harmful (46, 55). In turn, lower cannabis risk perceptions have been linked with higher rates of cannabis use (10, 89). While cannabis has demonstrated health benefits in limited medical situations (e.g., reduced nausea for cancer patients; 8, 92), cannabis use can have negative health impacts. For example, cannabis can impair motor function (40), and co-use of cannabis and alcohol is associated with greater risk of auto collisions (6, 14). Secondhand cannabis smoke raises the levels of psychoactive elements [i.e., tetrahydrocannabinol (THC)] in nonsmokers’ blood, including that of children (51, 111), and may have cardiovascular effects comparable to environmental tobacco smoke (108). Despite these risks, public polling shows that substantial proportions of US consumers either are unaware of or underestimate the health risks of cannabis (54, 67), which highlights the importance of informing the public about them (110).

**Figure 2:**
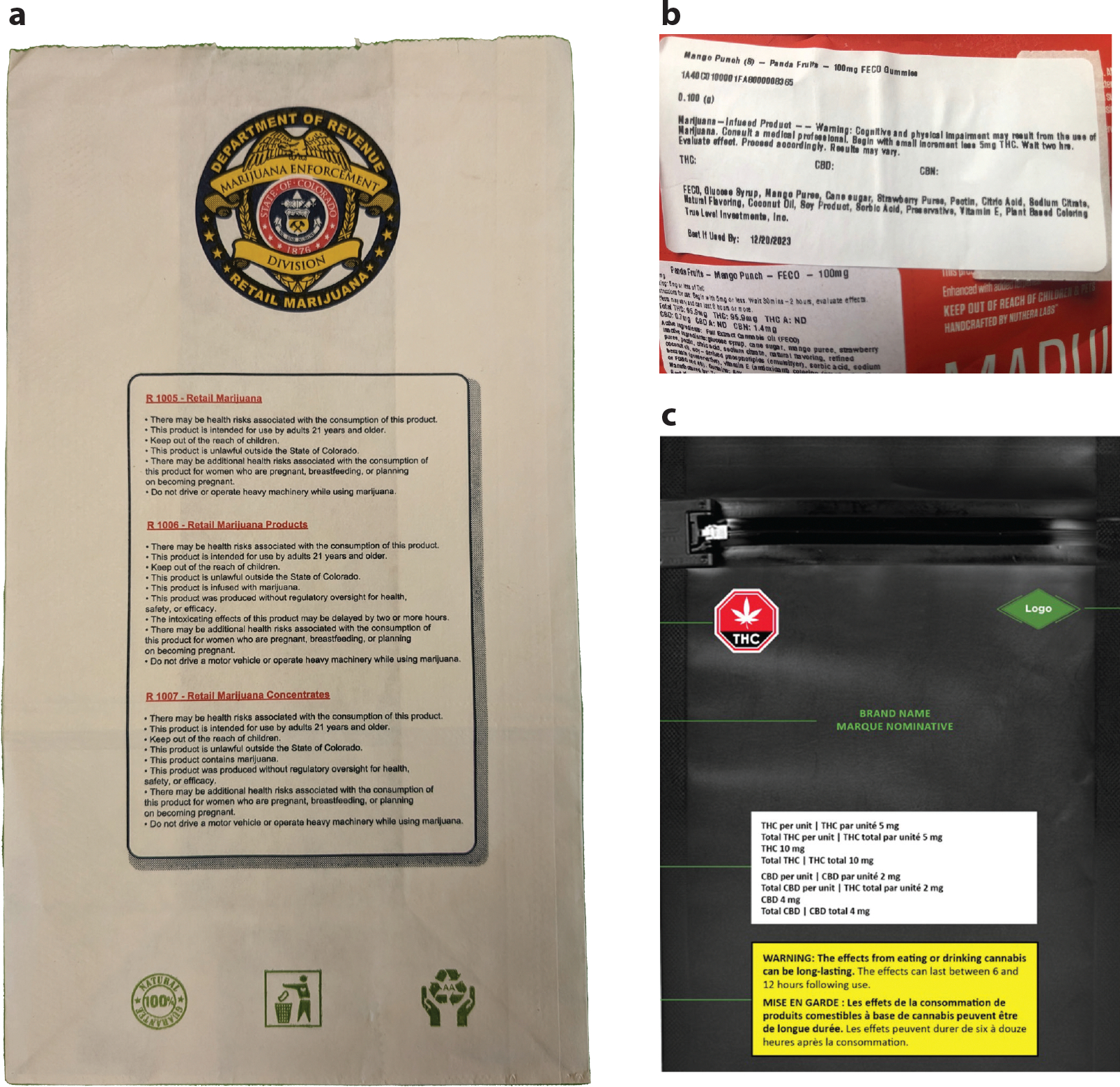
Cannabis warning labels from (*a*) Colorado, (*b*) Missouri, and (*c*) Canada. Canada (like Uruguay) requires cannabis products to be sold in plain packaging. Panel *c* reproduced with permission from Health Canada.

As more jurisdictions legalize recreational cannabis, they are adopting lessons from tobacco health warning research. For instance, Canada requires cannabis packaging to display a universal cannabis symbol, THC and CBD (i.e., cannabidiol) information, and text-only health warning messages about potential harms (Figure 2) (36). The warning messages address topics such as general risks (e.g., worsened mental health), risks from specific products (e.g., edibles), or risks for priority populations, such as adolescents and pregnant women (35). The warning messages must be printed in black text on yellow background with font sizes equal to the brand name. In addition, the warnings must be displayed prominently and apart from product information (36). Canadian regulations for labeling and packaging are arguably the most comprehensive consumer cannabis regulations worldwide (43).

In the United States, the legal status of cannabis and cannabis policies at the federal and state levels differs. To date, cannabis is a prohibited Schedule 1 substance at the federal level in the United States (105). However, recreational cannabis has been legalized in 23 US states. In addition, while all US jurisdictions allowing recreational cannabis require health warnings on packaging, the content, size, and placement of the warnings vary by jurisdiction (3, 11, 91). For example, policy analyses of cannabis warning labels from the first four US states to legalize recreational use (e.g., Colorado, Washington, Oregon, and Alaska) found that labels varied by state, but most labels failed to meet standards consistent with protecting public health, such as large pictorial warnings on the front and back of packaging, as recommended by the World Health Organization’s Framework Convention on Tobacco Control (5, 79, 80, 109). Given the legal uncertainty surrounding cannabis in the United States, no comprehensive regulatory framework has been adopted for cannabis warning labels.

There is an ongoing scholarly debate about the best way to inform consumers about the risks of using cannabis. Some scholars have argued for approaches informed by tobacco control policies, including mandating graphic warnings on plain packaging (2, 5, 79, 80). Others have argued for milder warnings that would focus on responsible use, similar to warnings on alcohol products (74). Both sides agree, however, on the need for research on best practices to inform consumers about the health effects of cannabis, especially as cannabis becomes increasingly available as a retail product (56).

### 3.1. Real-World Effects of Cannabis Warnings

In tobacco research, studies evaluated the effects of new or strengthened warnings on behavior (77). Because cannabis warnings in most jurisdictions came into effect alongside legalization, to our knowledge, there have been no studies on the effects of cannabis warnings on behavior. However, studies examined key precursors to behavioral effects, specifically whether a consumer notices and recalls labels, in the real world.

The International Cannabis Policy Study (ICPS) is a quasi-experimental study using repeated cross-sectional surveys to assess cannabis-use outcomes in Canada and the United States (44). A population-based ICPS survey of 16–65-year-olds in Canada and the United States (illegal and legal jurisdictions) was collected in 2018, before Canadian cannabis legalization, and again in 2019. Canadian respondents reported an 8.9% increase in levels of noticing cannabis warnings during that period, from 5.8% in 2018 to 14.7% in 2019 (33). Canadian warnings included mandated features (e.g., description of specific harms, black text on yellow background, prominent font).US jurisdictions showed modest increases in noticing from 2018 to 2019, with respondents in legal US jurisdictions reporting a 3.2% increase (13.9% to 17.1%) and those in illegal jurisdictions showing a 2.8% increase (5.9% to 8.7%).

Another ICPS survey of 16–65-year-olds from Canada and the United States (legal and illegal jurisdictions) measured free recall of cannabis warnings in 2018, 2019, and 2020. The analytical sample was current cannabis consumers (i.e., past 12 months) who were asked if they recalled seeing cannabis warnings on products in the past 12 months. Those who answered “yes” were asked to describe the warnings, and open-ended responses were coded for accuracy. For the Canadian respondents, accurate free recall of at least one cannabis warning increased from 5% in 2018 (prior to legalization) to 13% in 2019 (postlegalization) and to 15% in 2020 (34). The results from these international population-based surveys indicate that mandating warnings on consumer cannabis products was associated with higher levels of notice and free recall, both key antecedents for effects on behavior.

### 3.2. Testing Features of Hypothetical Cannabis Health Warnings

Research also continues to evaluate features of cannabis warnings to determine best practices for warning design. These studies typically develop hypothetical warning labels to gauge reactions to different warning features, such as warning themes or format (pictures versus text).

#### 3.2.1. Warnings themes.

Several studies in Canada and the United States exposed participants to warnings with different themes and assessed perceived message effectiveness, most frequently measured as believability and sometimes as the extent to which the warning made one think about reducing cannabis use (62, 72, 82, 112). Warnings about health harms were rated as most believable: for example, cannabis harms for pregnancy (62), brain development (72, 112), or impaired driving (82). In contrast, warnings focused on addiction (62, 72, 82, 112) or psychosis (82) were rated as least believable. Moreover, participants who saw any warnings rated cannabis smoking as more harmful than the no-warning condition, indicating that warning exposure impacted risk perceptions (82). Of note, the warnings used in these experiments were adapted from existing Canadian (62, 72, 112) or US warnings (82).

#### 3.2.2. Pictures versus text.

Tobacco research shows that pictorial health warnings are more effective than text-only warnings in impacting attitudes and behavior (42). Several studies compared the effects of pictorial and text-only warnings for cannabis. For instance, an experiment in Canada in 2017—one year before legalization—assigned 16–30-year-olds to view hypothetical pictorial (versus text-only) cannabis warnings (62). Warnings in the experiment were black text on a yellow background and described different harms (e.g., harms of impaired driving, smoke toxicity, harm to mental health). Pictorial warnings (versus text-only) were rated higher on perceived effectiveness and believability—two key constructs for developing warning labels (76, 78)—and this finding was consistent across different portrayed harms.

An experiment conducted in 2020 with 18–26-year-olds in the United States found that a warning that enhanced the existing Californian warning with a picture resulted in greater negative emotions, greater accuracy in recall of the warning information, and greater perceived message effectiveness compared with either the mandated California text-only warning or a mock text-only warning that added text about mental health to the existing Californian warning (58).

A 2017 discrete choice experiment of adults (age 21+) living in US states with legal recreational cannabis exposed participants to hypothetical cannabis products with randomly varied warning types (e.g., no warning, text-only warning, pictorial warning about impaired driving, and FDA disclaimer about cannabis) (93).The warning content was derived from Colorado and Washington state text warnings and adapted FDA content about cannabis harms. Notably, the driving warning was the only one with an image, and the warning read “Drive High: Get a DUI.” Results found that participants (never and past-year nonconsumers) would be more likely to purchase cannabis products with pictorial warnings compared with no-warning attributes. The study authors noted that this counterintuitive finding could have been due to the novelty of the warning with an image or that the image was not graphic enough to dissuade the purchase.

Overall, research on warning labels for cannabis products is growing but so far has mostly examined only limited outcomes, such as noticing and remembering warnings in real-world studies and perceived effectiveness (e.g., how believable and convincing the warnings are) instead of actual effectiveness in controlled experiments. In addition, the content of cannabis warning messages—particularly in hypothetical studies—varies widely, and results should be interpreted with that variation in mind. Future studies need to investigate the effects of warnings on perceptions of harm, addictiveness, attitudes toward the products, behavioral intentions, and, ultimately, behavior and need to examine warnings on various cannabis products (e.g., flower, vape liquids, edibles).

## 4. OPIOID MEDICATIONS

Opioids refer to a class of drugs with pain-relieving therapeutic, as well as euphoric, effects. While international efforts have been made to decriminalize and legalize the sale of some forms of opioids, commonly referred to as illicit or street drugs (e.g., heroin), this article focuses on the labeling of opioid medications for medicinal purposes (e.g., hydrocodone, morphine, methadone, oxycodone, fentanyl) (22, 38). The distribution, packaging, and marketing of opioids are highly regulated worldwide. This section examines evolving efforts to enhance patient understanding of the effects and risks of opioid medications when dispensed using warning labels in combination with educational materials and packaging.

### 4.1. Changes to Labeling, Packaging, and Distribution

Opioids hold a valuable place in the history of medicine, yet historical trends and the current opioid overdose crisis indicate that opioids are utilized for more than their medicinal purposes despite regulatory efforts to control their distribution and use. Opioid medications are valued for their ability to attenuate the body’s pain response and are often sought outside of clinical settings for their euphoric benefits. As a result, unregulated exposure to opioids has been linked to increased potential for misuse throughout history. A 1924 US public health report surmised, “It is believed that the trend of addiction in this country for the past six decades has paralleled very closely the quantities of narcotics available, as represented by the average annual importations” (60, p. 1197). Today, just as it was more than a century ago, rising opioid consumption has catalyzed labeling reform responses from government agencies, particularly in the United States. US opioid prescribing peaked in 2012, with substantial variation in dispensing persisting today across counties and states. In the last 24 years, more than 600,000 individuals in Canada and the United States have died from opioid-involved overdose (53). While opioid medications are not the main driver of the current opioid overdose crisis, they remain a key component, presenting in the toxicology of 16,706 fatal opioid-involved overdoses in 2021 (73). The excess use of opioid medication needs to be curbed while enhancing patient understanding of risks through various avenues, including medication labels and packaging.

Iterations of US legislation have sought to curb access to opioids outside of medical settings and to enhance labels for better transparency and patient understanding of risks. Key changes to opioid labeling to inform American consumers came in 1906 under the Pure Food and Drug Act, which required opium and derivatives to be labeled and listed on medications and consumer products (85). In tandem with increasing transparency through labels, regulators actively worked to reduce exposure to opioids. The Harrison Narcotics Tax Act, implemented in 1915, required prescriptions from medical providers for opioids to be dispensed to patients and mandated recordkeeping for physicians and pharmacists who dispensed opioids (16). This approach aimed to remove access to opioids through commercial markets. Over time, legislation specified that when prescriptions were filled, the package labels must include the dispenser’s name and registry number; the serial number of the prescription; the name and address of the patient; and the name, address, and registry number of the practitioner issuing the prescription. Further regulation over the twentieth century in the United States, including the Federal Food, Drug and Cosmetic Act of 1938, the Durham-Humphrey Amendment of 1951, the Kefauver-Harris Amendments of 1962, and the Controlled Substances Act of 1970, required specific language to be included on opioid labels, reading “Warning—May be habit forming” and “Caution: Federal law prohibits dispensing without prescription,” as well as an indication for the use of medication and side effects; however, it provided no guidance on where or how that information should appear (26,52,97,99).Regulation of opioid labeling and marketing continues into the twenty-first century.

### 4.2. Current Requirements for Medication Warnings

**Figure 3:**
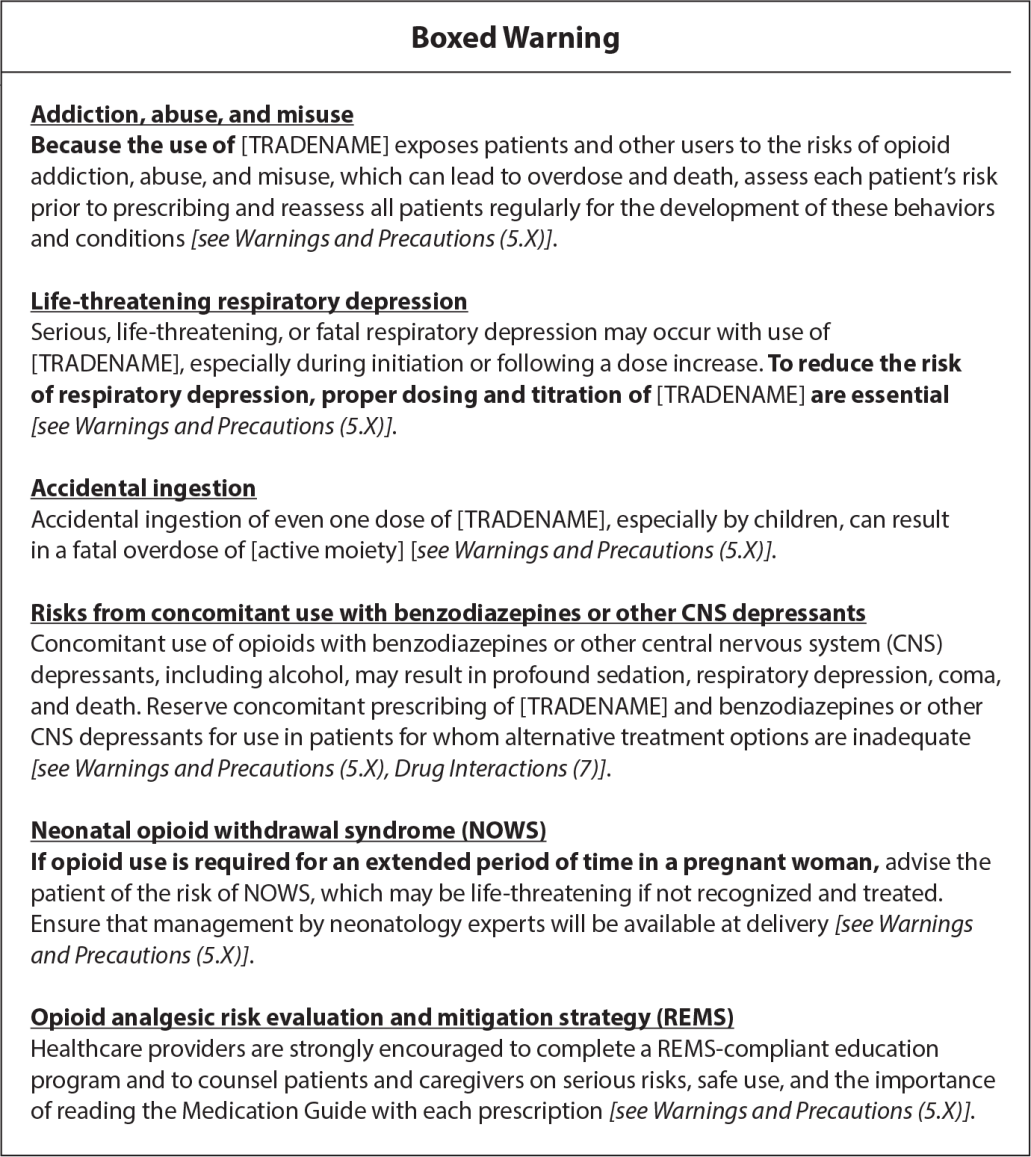
FDA boxed warning for immediate-release and extended-release/long-acting opioid analgesics’ packaging and inserts (from https://www.fda.gov/media/167056/download).

Regulators continue to institute labeling changes and warnings on opioid medications. Boxed warnings, also known as black box warnings, are the highest, boldest safety-related warning required by the FDA on opioid medications, which seek to bring consumers’ awareness to major risks (37). Standardized class-wide language is required to be used on boxed warnings to communicate to consumers that opioids hold serious risks of misuse and abuse, which can lead to addiction, overdose, and even death (Figure 3) (28, 30). Indications for prolonged use of extended-release and long-acting opioid medications outlined on prescription medication inserts have been narrowed. These indications clarify that opioids carry risk for the development of neonatal opioid withdrawal syndrome when taken during pregnancy and opioid-induced hyperalgesia (e.g., increased pain and sensitivity) (17, 28, 30). Box warnings on package inserts advise patients of interactions with central nervous system depressants (e.g., benzodiazepines) and how tablets should not be broken, chewed, crushed, or dissolved as doing so may cause rapid release of the drug, which could be fatal. The distribution, labeling, and dispensing of opioid medications have evolved to enhance transparency and communicate an ever-lengthening list of warnings. As a result, warnings are often found in medication packaging or patient leaflets rather than on the label. Little is known about how effective these approaches are in enhancing patient understanding of medication uses and risks.

Similar to regulations for warnings on other substances (e.g., tobacco), medication labels, packaging, and marketing-related regulations have been subject to influence by special interest groups and manufacturers. One of the most egregious examples was OxyContin. When this medication first appeared on the market, the producers ensured that its packaging stated that iatrogenic addiction was very rare if opioids were used in the management of pain. This false narrative was echoed in their marketing materials (106). FDA labeling mandates have since evolved from diminishing risks for addiction to now communicating risks and actionable solutions for mitigating poor outcomes. The FDA recently mandated that opioid medication packaging include a recommendation that patients and caregivers discuss with their clinician whether naloxone, a potentially life-saving opioid-involved overdose reversal medication, should be obtained (29). The number of warnings on opioid medications continues to expand, yet government agencies provide little guidance on how information should be presented to impact patient outcomes.

There has been little evaluation of how changes to opioid medication labels and packaging have shifted prescribing and overdose trends. The FDA requires manufacturers of extended-release and long-acting opioid medication to provide voluntary continuing education for prescribers on the Risk Evaluation and Mitigation Strategy and to create medication guides to inform patients about opioid medication risks (23). Research has been inconclusive on whether these efforts reduce inappropriate prescribing or improve patient outcomes (48, 88). Education is not mandated for prescribers and dispensers on effectively communicating to patients about the indications and risks of opioid medications. Empirical evaluations of governmental efforts to enhance patient understanding of the safe medical use of opioid prescriptions through packaging and accompanying educational materials to curb the ongoing global opioid overdose crisis are warranted.

### 4.3. Challenges to Communicating Medication Risks

Manufacturers and dispensers must meet government labeling standards worldwide, yet there are no consistent guidelines on the most effective labels. Regulators require black box warnings on certain medications, including opioids, that highlight contraindications, precautions, and warnings. However, the location, length, and contents of warnings differ by country (9, 57, 94). The optimal medication warning label is well-designed, clearly states patient-centered medication information, and can enhance patient understanding of appropriate use (52). Research shows that written instructions in large font, at a fifth-grade reading level, with information grouping, sufficient white space, and typographic cues (e.g., bullet points, bold font) are most useful in communicating risks, dosing, and benefits to patients (71). Results concerning the use of icons and containers for medications have been mixed (71, 95). Barriers to patient understanding persist, including health literacy, consumers being dispensed multiple medications, language proficiency, and vision impairment (4, 24). Despite these research efforts to improve how patients consume information on medication labeling, few studies on enhancing opioid-specific labels have been conducted.

Most opioid-dispensing patient-centered research has been on supplementing label and packaging information with enhanced patient education. Providing patient-centered educational materials and follow-up text messages showed mixed success in enhancing patient understanding of opioid-related risks compared with labels alone (65, 66). Rather than improving opioid medication labels, interventions have centered on increasing naloxone dispensing and the disposal of excess medication to prevent misuse, overdose, and death through additional supplementary written patient-education interventions, personalized communication via text messages, and multimedia campaigns that communicate opioid-related risks using narratives (1, 13, 32, 68). Regulators continue to expand the number of warnings required on opioid medications with little guidance on how to effectively communicate these new risks to patients. Simultaneously, researchers persistently test time-intensive and not necessarily easily scalable patient-centered educational interventions that do little to optimize the physical labeling, packaging, and dispensing of opioids to patients.

Significant barriers remain to placing effective warnings on opioid medication labels. All medication labels face a challenge to prioritize text on limited label real estate, with much of the required language on medication safety crammed onto package inserts and patient information leaflets (31, 96). Many patients do not read instructions on package inserts, and those who do find the information hard to understand (113, 114). As many as one in two patients find medication labels difficult to read and report not understanding medication risks based on their reading of the labels (83). Most medication labels use very small fonts and overemphasize unnecessary information, such as the pharmacy logo (95). Limitations in most pharmacy-based printer capabilities (e.g., color, fonts) further hinder opportunities to redesign label content and incorporate icons or pictograms. The need for standardized patient-centered labeling on opioid medications remains a persistent challenge in high-volume pharmacies that need to efficiently dispense numerous classes of medications.

### 4.4. Opportunities to Enhance Labels and Dispensing

Opioid medication labels and inserts are a major source of vital information on benefits, risks, and safe use for patients and their caregivers. This vital information is often inconsistent across dispensing locations and is difficult to comprehend by patients (96). Changes to opioid medication warnings continue to evolve as regulators seek to include the most up-to-date and pertinent information on potential risks and solutions to mitigate poor outcomes, yet limited space on labels and packaging, in tandem with a lack of patient comprehension of and engagement with the dispensing materials, creates problems. Research on how evolving changes to warnings impact patient outcomes is needed. While best practices for enhancing label and package insert readability for patients are well established, less information exists on how they apply to opioid medications. Using personalized communication and audiovisual tools shows increasing promise for enhancing safe use, risk mitigation, and disposal of opioid medications; however, future clinical trial research is needed with opioid medication warnings, as have been conducted with tobacco and cannabis.

Concerted efforts have focused on developing tamper-resistant, or abuse-deterrent, formulations of opioids that could be marketed and labeled as “safer” than traditional immediate and extended-release medications (69). Safety language on labels echoes comparative risk messaging being implemented in tobacco products. It remains unclear how enhanced safety benefits of these emerging medication formulations would be received by patients. Internationally, clinicians and regulators recognize the importance of opioid medication warning labels as an opportunity to ensure safe use of prescription opioids by patients (64). Developing and implementing effective labeling, packaging, and accompanying dispensing strategies capable of overcoming known challenges to patient understanding and communication of mandated warnings remain elusive.

## 5. CONCLUSIONS

This article has reviewed the history of developments in and the current state of warning labels for three substances: tobacco, cannabis, and opioid medications. Tobacco warning labels have the longest history and provide the template for research and policy in other areas. The tobacco industry’s resistance and legal challenges to cigarette warning labels indicate that the warnings passed the “scream test” (20) and are an effective tobacco control measure. For novel tobacco products (e.g., e-cigarettes) or cannabis, if the science demonstrates evidence of serious health harms, warnings need to be developed that communicate these harms and that are in line with the standards of effective warnings (large pictorial warnings that cover both the front and back of the pack, in the language of the country, and frequently changed to avoid a wear-out effect) (109).

Warning labels exist not in a vacuum but in tandem with policies, public education campaigns, and other interventions. Such is the case for all three substances reviewed here. While it is important to continue developing more effective warning labels and working to implement them (as is the case with cigarette pictorial warnings), in the larger scheme of things, it might not matter as much, particularly in the tobacco landscape. In the United States, the reduction in smoking rates has been a tremendous public health success, with smoking rates at 11.5% for adults in 2021 (15). This decrease has been achieved with old warnings in combination with smoke-free policies, taxes, communication campaigns, and other efforts to denormalize smoking. However, the rates might have been even lower had the pictorial warnings been implemented on schedule. Estimates show that a 10-year delay in implementing the pictorial warnings might have cost ~179,000 lives (100). Because implementation is not in sight yet, this number will likely grow.

Findings from tobacco labeling research are being integrated into the labeling of other substances, notably for cannabis. Evaluation efforts are needed to discern future potential public health benefits from labels in communicating and mitigating risks among consumers. Given the dynamic and uneven regulatory landscape for retail cannabis products in the United States, it is unclear how variations in cannabis product labeling may influence use behaviors across jurisdictions. The diverse forms of cannabis products (e.g., flower, vapes, edibles, drinks, lotions) and an uncertain legal environment highlight the need for more research to determine how to effectively communicate risks to consumers.

Aggressive marketing from manufacturers and special interest groups has resulted in a need to effectively communicate the risks of these substances to consumers in succinct and effective ways. Future research examining the effects of warnings on perceptions of harms, addictiveness, attitudes toward the products, behavioral intentions, and, ultimately, behavior across cannabis products and opioid medications is needed. Research must then be translated into practical labeling changes from regulatory agencies. Research on opioid-related risks over the past two decades has resulted in an increasing number of warnings attached to opioid medication labels and inserts and in narrowing indications for use. Despite the increasing length of warnings, little research has examined how patients consume and utilize this information on their medications or how these labels could be formatted to mitigate risks. As the number of cannabis consumers increases and prescription opioid-involved overdoses persist, optimized labeling presents an effective and scalable public health intervention.

Effective warning labels on substances must clearly communicate risks to consumers. In the case of cannabis and opioid medications, there is an additional need for labels and product language to indicate proper use and approaches to reduce risks for adverse effects and poor outcomes. Special interest groups and manufacturers have hindered the implementation of evidence-based labeling approaches capable of achieving these goals. Continued investments in rigorously evaluating the benefits of labeling and packaging of tobacco, cannabis, and opioid medications for individuals, health systems, and communities are warranted.
